# Childcare and maternal part-time employment: a natural experiment using Swiss cantons

**DOI:** 10.1186/s41937-017-0003-x

**Published:** 2018-07-03

**Authors:** Laura Ravazzini

**Affiliations:** 1 University of Neuchatel, fbg. de l’Hôpital 27, Neuchâtel, Switzerland; 2 FORS, Géopolis, University of Lausanne, Lausanne, Switzerland

**Keywords:** Childcare places, Maternal employment, Policy evaluation, Quasi-natural experiment, Part-time, J13, J22, H40

## Abstract

Fuelled by federal stimuli of 440 million Swiss francs, the staggered expansion of childcare in many cantons allows the evaluation of this family policy on female labour supply. With new cantonal data, this study analyses both the decision to participate in the labour market and the intensity of participation. Empirical results of difference-in-differences regressions show that mothers work at higher percentage rates if they live in cantons that have expanded their childcare services more than the national average. The reform stimulated part-time employment of between 20 and 36 h per week by 2 percentage points. The expansion of childcare particularly affected women with two children and upper-secondary education, who are married or cohabit with their partner.

## Background

Many modern welfare states have prioritised assisting women in their work-family life. One of the tools used by policy makers to stimulate the labour supply of mothers is through the expansion of childcare. In countries in which female labour force participation is already high, the effects produced by childcare are usually minimal (Bettendorf et al. 2015). Recent studies in many Scandinavian countries indeed show that subsidised childcare has had no significant impact on female labour supply (Lundin et al. 2008; Havnes and Mogstad 2011b).^1^ As these studies suggest, this is probably because the services provided in these countries meet the demand for childcare among all social classes almost perfectly. This does not appear to be the case in Switzerland, where, as in Germany (Wrohlich 2008; Bauernschuster and Schlotter 2015), the demand for childcare is much higher than the supply (Banfi et al. 2009). In Switzerland, female labour force participation is now very high, with 79.8% of women actively participating in the labour market (OECD 2015). The participation of women with children, however, is much lower, and as in the Netherlands, the majority of this high rate of work force participation is part-time work. This finding suggests that childcare could potentially influence working hours rather than labour market entry itself.

The extensive margin of female labour force participation linked to the availability of childcare has been studied in many countries, and results appear to differ depending on the national context (see Cascio et al. 2015 and Del Boca 2015 for a review of recent studies). Evidence of the effects on the intensive margin is something that has been studied only recently. This paper contributes in this respect by examining a natural experiment of childcare expansion in Switzerland.

This paper analyses the effects of the increase in the number of childcare places driven by public subsidies, applying a quasi-experimental design in 24 Swiss cantons. The Swiss federal system is peculiar in its characteristics because each canton can legislate over many aspects that affect the socio-economic life of its population. This wide variety of legislation creates variability in the data, which becomes useful for evaluating policies. In addition to this political diversity, the Swiss context is interesting because many aspects of its welfare state have led it to be classified among liberal countries. Unlike modern Anglo-Saxon countries that have promoted women’s independence since the beginning of the twentieth century, the values and gender roles of this country have been traditionally anchored in the classical male breadwinner model. This means that the government intervenes only sporadically to regulate family matters and that social norms do not create strong work attachment among women.

In addition to this, many factors intervene in women’s decisions to participate in the labour market. These factors include the preferences of the individual and of the partner, culture and discrimination (Milligan 2014). Recent studies show that a gender egalitarian culture is still not widespread in Switzerland (Epple et al. 2015) and that decisions regarding paid employment for mothers are strongly influenced by their partner’s income (Gerfin and Leu 2007; Ernst Stähli et al. 2009). As highlighted by Asai (2015), in Japan, the participation of fathers in childrearing could substantially reduce mothers’ hardships in reconciling work and family life. In Switzerland, the influence of partner’s income on female labour force participation declines 4 years after childbirth, but not before (Liechti 2017).

In this context, the evaluation of the effect of childcare policies is not a trivial exercise. This is true for several reasons. First, when formal childcare places are rationed, the creation of new slots or the introduction of subsidies might simply substitute other non-institutional childcare arrangements among mothers who are already working (Givord and Marbot 2015). If this occurs, the political effort put into the expansion of childcare may appear unsuccessful (Havnes and Mogstad 2011b). Second, when most women prefer part-time work arrangements,^2^ it is difficult to measure full-time equivalents because women may use childcare at different rates. Analyses of the effects of an expansion of childcare places should therefore take into account these preferences and select a variable that can detect subtle changes in working hours. In addition to this, when most women work, changes are expected to be found more on the intensive margin than on the extensive margin. The expansion of childcare places in a country with high labour force participation and widespread part-time work translates into an expansion in the number of days per slot available. Third, prices can be a deterrent for the use of childcare among certain types of households. In this case, when prices are not directly available, it is important to test the assumptions on a broad spectrum of household types in order to have a reliable picture of the overall effects. Fourth, childcare services may be steered by parental demand for this type of service and by political interests based on the specific characteristics of local labour markets. If the supply is driven by demand, then estimates are likely to be biased. It is therefore important to correct for this possible endogeneity.

This study uses data from the Swiss Labour Force Survey (SLFS) and a newly established database with cantonal data on the number of childcare places in recognised private and public childcare facilities for early childcare. The identification strategy consists of a quasi-experiment created by different degrees of expansion of childcare places among 26 Swiss cantons between 2002 and 2012. The target population comprises mothers with at least one child between 0 and 3 years of age. A difference-in-differences analysis is used to evaluate the effect of the expansion of childcare places on the labour market outcomes of these mothers.

The results demonstrate that an expansion in childcare places stimulates part-time employment of between 20 and 36 h per week by 2 percentage points for mothers of 3-year-olds. The reform has not triggered any change in female labour force participation but has allowed working mothers to work longer hours. The effects appear greater for married or cohabitating mothers with upper-secondary education. Significant effects are registered for mothers of two children. No effects are found for fathers.

## Federal family policy and childcare expansion among cantons

The demand for childcare services in certain Swiss regions has been found to be extremely high, with up to 8.1 children enrolled in waiting lists for each childcare place (Banfi et al. 2009, Dasoki et al. 2011). To satisfy this demand, in 2003, some federal subsidies of 200 million Swiss francs were granted to early childcare and after-school facilities for a period of 8 years. This public incentive has been renewed twice for another 240 million francs, and subsidies are expected to last until 2019. By the beginning of 2016, the total allocated amount was nearly 300 million francs, and around half of it (133.8 million francs) was claimed by early childcare facilities. The subsidised childcare facilities had to be appropriate in terms of the ratio of trained educators per child and had to satisfy certain technical requirements and opening hours. This was done to guarantee high standards for the quality of care.

The allocated subsidies did not alter the prices that parents had to pay to enrol their children in these facilities, but they covered some fixed costs that could reduce the profitability of opening or expanding a childcare centre. The price that parents must pay for these childcare places depends on their total household income and the municipality in which they live. As previously stated, this price remains high by international standards (Stern and Felfe 2015).^3^ Among high-income families, it becomes financially inconvenient for the household to rely on external childcare if the second earner works more than a part-time rate of 60% (Bütler and Ruesch 2009). The price of childcare is therefore a strong disincentive for female labour supply, and this disincentive seems particularly high for low-income families (Abrassart and Bonoli 2015). Childcare is indeed expensive, but women—and particularly highly educated women—may react more to childcare availability than to prices when the market of childcare services is rationed (Kreyenfeld and Hank 2000; Del Boca and Vuri 2007; Bassok et al. 2014). This is, of course, something that should be tested with accuracy in the framework of this study.

Overall, these financial incentives boosted childcare and after-school places in both public and private facilities.^4^ Over a period of 13 years, the supply increased by 28,480 places in early childcare facilities and by 22,121 places in after-school facilities. Despite this success, almost half of childcare facilities declared that they could not satisfy the demand due to inadequate time slots, which in most cases meant that there were fewer places than the demand for those places on certain days of the week (OFAS 2016). Given the high share of women working part-time in Switzerland, it is not surprising that women need childcare slots on some days more than others. Even if the reform has expanded the number of days per slot available, this expansion still seems far from meeting the demand. Additionally, some peripheral regions have not seen notable gains from this expansion (Schmid et al. 2011). Political priorities, intentions to attract highly qualified migrants to the area, and the availability of infrastructure and of childcare providers are some of the reasons that may have led to differences in the implementation of childcare services. Regardless of where the facility is based, it is the responsibility of the (new) childcare facility to request the subsidy. This fact may therefore reduce the importance of cantons in determining the demand for childcare.

It is also interesting to note that childcare was not the only family policy introduced in the early 2000s. In 2005, maternal leave was established, providing a federal minimum of 14 weeks at 80% of normal salary. This federal legislation was largely a legal confirmation of what the vast majority of employers had established in the early 1990s. Since it was designed in many ways to set a minimum for special cases (e.g., women in their first year of employment), paid maternity leave should be seen as an accompanying measure to childcare, and not as a major reform. The experiences of other countries also show that job continuity is not affected if leave entitlements are for up to 17–18 weeks (Baker and Milligan 2008), which is much longer than what has been legally established in Switzerland. On top of that, some cantons reformed their constitutions to establish a legal enforcement of after-school care. As illustrated by Felfe et al. (2016), this legal enforcement, however, had an impact only in the German-speaking part of Switzerland, where after-school care was particularly underdeveloped.

Apart from these measures, home care leave or paid childcare leave after parental leave do not exist, and while some cantons allow around 2 weeks of paid paternal leave, paternal leave is not mandatory at the federal level.

## Methods

### Data

This analysis uses data from the Swiss Labour Force Survey of the Swiss Federal Statistical Office. This is an annual survey that focuses on the employment situation of the permanent resident population aged 15 and older. The survey includes labour supply indicators (participation, income, part-time rates), socio-demographic characteristics (marital status, age, education, nationality) and some limited information about other household members (total household income, age and education of each household member). Repeated cross-sections begin in 1991, and this allows for the study of the pre-reform period for both the targeted group and the control group.^5^ A special module on childcare is included every year from 2001 to 2010, as well as in 2013. However, the wording of the question changed in 2010, which made it possible only to compare the answers between 2001 and 2009.

Data on the number of childcare places come from a newly established questionnaire sent to each cantonal administration to collect relevant information about the evolution of childcare services from 1991 to 2012. More information about these data can be found in Ravazzini et al. (2016). Data from the 1990s is extremely scarce, because very few cantons had electronic archives. Only in the 2000s did information about childcare places become more accurate. Overall, 24 out of 26 cantons have information about the number of childcare places in their public and private facilities for at least 2 years around 2002 and 2012.^6^ If data for these exact 2 years are not available, information is taken from the years closest to those dates, making the reliable assumption that childcare places do not change radically from 1 year to the next, but rather over subsequent decades.^7^ One of the limitations of the data is the lack of detail at the municipal level, which is important in the Swiss context, because municipalities are sometimes more competent than cantons in regulating childcare activities.^8^ Another limitation of the data is the lack of information about the price of external childcare. To overcome this problem, the analysis is run for several household types. Moreover, even if it is clear that the federal government authorises and subsidises only those childcare facilities that meet certain standards, there is no particular specification regarding the quality of childcare. This paper assumes that the quality of service in authorised childcare facilities is the same everywhere across the territory and that parents could substitute other forms of care with this one if they were able to do so. This assumption of uniform quality may not hold in countries such as Germany, where there is a large divide between the East and the West (Schober and Spiess 2015), but it should be a reliable assumption for a small country such as Switzerland.

### Methodology and sample

**Fig. 1:**
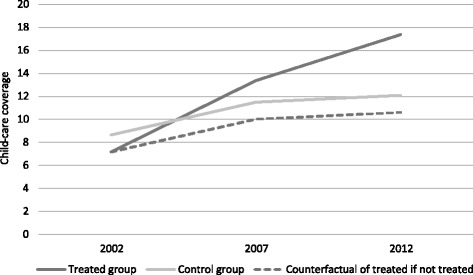
Evolution of childcare coverage, comparison between treated and control groups. Source: Administrative data on cantonal childcare places (Ravazzini et al. (2016), 2012 cantonal statistics for Bern, Neuchâtel and Vaud). The dotted line highlights the expected behaviour of the treated group in absence of treatment. A linear trend is assumed to interpolate the missing years

The relationship between childcare provision and female labour supply is identified using a difference-in-differences model (DiD) (see Imbens and Wooldridge 2009). This model compares the labour market outcomes of a specific group (treated) targeted by a political reform with the outcome of a similar group (untreated) that is untouched by the reform. The methodology consists of a comparison before and after the reform, under the assumption that the groups would have reacted in the same way without a reform. To rely on this assumption, the common trend is tested in the years preceding the reform. The groups treated in this paper consist of working-age mothers (between the ages of 20 and 50)^9^ who have at least one child up to 3 years of age. These mothers could particularly benefit from the expansion of childcare services if they lived in those cantons in which the expansion was higher than the national average.^10^ Since the allocated subsidies targeted mothers of children of different ages through both childcare and after-school services, it was not possible to exclude mothers of older children who might have benefitted from the reform. The administrative data collected by Ravazzini et al. (2016) do not include information about the number of after-school places. Mothers who use childcare services for more than one child can also benefit from special prices. The targeted group in the majority of the analyses in this paper is therefore composed of mothers who have at least one child up to 3 years of age and the possibility to have also older children (up to 15 years old, as indicated by the policy). The control group consists of the same type of mothers who reside in cantons in which the expansion was lower than the national average. This definition of treated and control group is the same one used by Havnes and Mogstad (2011b) for Norway and by Bauernschuster et al. (2015) for Germany. It is important to remember that, in this empirical setting, all cantons were touched by the reform, but some cantons were more active than others in expanding their childcare places (see Table 9 in the Appendix). The evolution of childcare coverage between the treated and the control groups is represented in Fig. 1.

From Fig. 1, it appears that the net increase in coverage consists of 10 percentage points over 10 years, a small increase compared to those produced by other similar reforms carried out in Europe (in Spain, for instance, the net increase was 25 percentage points over 6 years).

The econometric model for the difference-in-differences used in the analysis could be written as:

1 $$ {Y}_{it}=\alpha +\beta {P}_t+\gamma {T}_i+\delta\ {PT}_{ti}+\rho {X}_{it}+{Z}_c+{Y}_t+{\varepsilon}_{it c} $$

where *Y*_*it*_ is the outcome variable. Given the importance of analysing the extensive and the intensive margins separately, this paper uses three different outcome variables: active participation in the labour market at any participation rate, participation at high part-time rates (> 50%), or participation only at full-time rates (90–100%).^11^ The reference group changes according to the three outcome variables; it is composed of inactive mothers in the first case, inactive mothers and mothers who work at low part-time rates in the second case, and inactive mothers and mothers who work part-time in the third case. This definition of the dependent variable allows the creation of progressive employment statuses without restrictions on participation, which, in this case, could be endogenously determined. In Eq. (1), *P* indicates the years prior and post the reform, which are 1992–2002 vs. 2003–2014, and *T* the affiliation to the treatment or to the control group. The intention to treat effect is estimated by the coefficient *δ*. Canton (*Z*_*c*_) and year (*Y*_*t*_) fixed effects are also added, and standard errors are clustered accordingly in order to correct for the specificities of each canton. Personal characteristics (*X*_*it*_) are included in the model to control for possible time-varying influences on the outcome variable. These personal characteristics consist of education, income of other family members, age, nationality and number of children. A further disaggregation of the results is performed according to the educational level of the mother. Table 1 shows that labour force participation is strongly dependent on the educational level of individuals, among other socio-demographic characteristics.

**Table 1:** Participation in the labour market according to individual and household characteristics

Characteristics	Maternal labour force participation	Share	Paternal labour force participation	Share
Educational level				
Lower secondary	41.34	9.69	87.96	11.04
Upper secondary	68.97	46.90	94.52	38.36
Tertiary	75.97	43.41	97.47	50.60
Marital status				
Single parent	74.87	17.03	90.98	15.02
Married or cohabitating	67.78	82.97	96.05	84.98
Number of children				
Small family (1–2 children)	70.16	84.79	94.94	83.01
Large family (3+ children)	62.45	15.21	96.96	16.99

Source: Swiss Labour Force Survey, 2014

The sample is restricted to mothers and fathers between 20 and 50 years of age with at least one child up to age 3

Low-educated fathers have a labour market participation rate that is almost 10 percentage points lower than high-educated fathers. The difference between low- and high-educated mothers is even more dramatic, at more than 34 percentage points. In an analysis based on the effects of childcare, Stadelmann-Steffen (2007) confirms the importance of this distinction by educational levels, demonstrating that the heterogeneous effects of regional childcare policy in Switzerland mainly affect the behaviour of women with an intermediate education level. Because her analysis used the number of childcare facilities in 2001, which differs from the number of childcare places, her results are not necessarily expected to match those estimated in this analysis. After the introduction of childcare subsidies in 2003, many existing institutions expanded their offerings. For this reason, we see more of an increase in the number of places in each childcare institution than in the number of institutions.

The possible effects of expansion of childcare places on fathers are also tested. Robustness checks of Eq. (1) include changes in fertility decisions, inter-cantonal mobility and the use of institutional childcare. Two tests are also performed to estimate possible confounding effects due to the almost simultaneous policies introduced in this period, such as the introduction of paid maternity leave and the legal enforcement of after-school care. The main DiD is further tested with two alternative specifications. The first specification excludes years that are too close to the introduction of subsidies. This is done because the expansion of childcare places may have produced nonlinear effects in the years before and after the first introduction of childcare subsidies. To exclude nonlinearities, the years are restricted to 7 years (1992–1998) for the period before the subsidies and to another 7 years (2006–2012) for the period after the subsidies. The years after the last data collection on childcare places are intentionally excluded (2013 and 2014). The second specification is inspired by the method applied by Felfe et al. (2016). This method controls for endogeneity of demand-induced supply through the construction of local labour markets. According to this innovative methodology, only some municipalities are retained in the analysis in order to gain a comparable set of treated and control cantons. These municipalities are situated inside a spatial mobility area composed of at least two neighbouring cantons and have similar voting preferences with regard to family policies.^12^ This procedure assures that the restricted sets of municipalities constituting the cantons are comparable in terms of labour market opportunities and of attitudes towards family policies. Cantons that have the majority of the population in the selected municipalities are omitted from the analysis, and this further excludes eight small cantons. As in Felfe et al. (2016), data regarding spatial mobility regions and votes for the 2006 referendum on family benefits are retrieved from the Swiss Federal Office of Statistics.

## Results and discussion

### Descriptive results on maternal employment

Treated and control cantons do not differ greatly in terms of the socio-demographic characteristics of mothers. Table 2 shows how the sample differs between these two groups before and after the expansion of childcare places. Significant differences between control and treated cantons prior to the reform can be found in educational level and in Swiss nationality. The treated group has a slightly less educated composition of mothers than the control group, whose mothers are more likely to be Swiss and to be more educated. This socio-demographic composition is unlikely to explain the expansion of childcare places in either of the two groups of cantons. As a general trend that can be observed in all cantons, the table shows a clear expansion in those with tertiary education, signalled by the twofold increase in mothers with a tertiary education before and after the introduction of childcare subsidies. Mothers have also become older. This comes as no surprise, as official statistics report that the average age of women at birth of first child has increased from approximately 29.1 in 1992 to 31.7 in 2014. About half of the sample has at least one child between 4 and 15 years of age in addition to at least one child between 0 and 3 years of age. In terms of other socio-demographic characteristics, the number of married women declined by approximately 6 percentage points, but marriage remains the preferred choice among mothers (more than 85% are married in the post-reform period). Mothers with Swiss nationality represent the majority of the sample, even if the percentage is slightly decreasing due to recent immigration and different fertility rates between the native and foreign-born populations. The share of single mothers among the sample also slightly increased over time. Differences in the socio-demographic composition of cantons and fertility rates after the reform are the object of two robustness checks that are performed later in the results.

**Table 2:** Sample description of mothers with children between 0 and 3 years of age before and after the reform

Year	Total	Treated canton		Control canton	
		Before	After	Before	After
Maternal age					
20–30	36.70	41.07	30.67	43.48	31.05
31–40	58.27	55.87	62.40	53.78	61.68
41–50	5.03	3.06	6.93	2.74	7.27
Maternal education					
Lower secondary	15.30	18.26†	14.89†	16.06†	13.23†
Upper secondary	61.80	66.87†	55.23	69.60†	55.10
Tertiary	22.90	14.87	29.88†	14.34	31.67†
Number of children under 4 years old					
1	80.09	80.72	79.65	80.16	79.93
2	19.08	18.34	19.44	19.07	19.31
3 or more	0.83	0.94	0.91	0.76	0.77
Number of children aged 4 to 15					
0	54.19	51.47	55.91	53.58	55.34
1	31.98	32.70	30.77	32.05	32.15
2	11.21	13.22	10.73	11.59	10.05
3 or more	2.62	2.60	2.59	2.78	2.47
Married	89.35	92.35	86.66	92.91	85.79
Swiss	69.14	71.03†	62.26†	75.07†	66.03†
Single mothers	2.60	1.95	3.34	1.85	3.29
Income of other family members	74,319	64,508	72,044	72,456	82,384
*N* (unweighted)	43,519	4567	11,283	7062	20,607

Source: Swiss Labour Force Survey 1992–2014

All figures are weighted percentages of the corresponding subsample except for the income of other family members and the number of cases. Income of other family members is represented in Swiss francs. The sample is restricted to mothers between 20 and 50 years of age

†Significant differences at 1% level between treated and control cantons

**Fig. 2:**
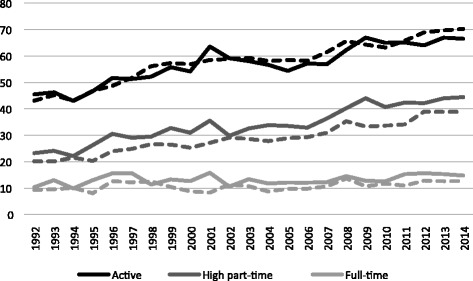
Change of several dependent variables on maternal employment over time depending on the canton of residence. Source: Swiss Labour Force Survey 1992–2014. The dotted lines indicate control cantons and full lines treated cantons

In terms of labour market outcomes, the two groups again show very similar trends (Fig. 2). Maternal labour force participation has increased enormously over the last 20 years. While less than half of mothers worked in 1992, almost three quarters did so in 2014. This was mainly due to an increase in the amount of mothers working at high part-time rates, which means more than a part-time rate of 50% or more than 20 working hours per week. This Swiss distinction regarding part-time rates differs from the standard OECD definition that sets the threshold for full-time employment at 30 usual weekly working hours in the main job (which corresponds to 75% of a standard working week of 40 h). Therefore, a significant share of mothers who are defined as part-time workers in Switzerland would be included among full-time workers using a European definition. In this paper, national definitions are used in order to highlight the importance of part-time work among women in this country. Although this figure is purely descriptive and does not allow us to spot significant differences, mothers in treated cantons appear to work in high part-time jobs more than mothers in control cantons.

**Fig. 3:**
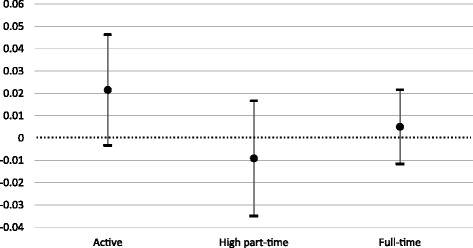
Estimates for the parallel trend assumption test. Source: Swiss Labour Force Survey 1992–2014. Coefficients are represented with their 95% confidence intervals. Insignificant coefficients validate the parallel trend assumption

These figures illustrate trends, but do not provide robust tests on whether the parallel trend assumption was satisfied before the introduction of subsidies. To observe whether the treated and control groups had parallel trends, a regression similar to the main DiD equation (Eq. (1)) is performed substituting the post-reform period with the pre-reform period (1992–2002). The results for the equivalent of the coefficient *δ* for the pre-reform period are illustrated in Fig. 3. As none of the coefficients are significantly different from zero, the parallel trend assumption should not be rejected. A test on the pre-reform period year by year yields significant coefficients for participation and full-time employment only in 2001. This confirms that the common time trend holds for all years for high part-time employment and for all years except for 2001 for labour force participation and full-time employment.

### Main results on maternal employment

Descriptive statistics alone do not provide a clear interpretation of the impact of the reform of female employment. The results of the estimates of the DiD model are required, and they are illustrated in Table 3, which is constructed illustrating yearly estimates first and then average effects over the post-reform period. The results of the first three columns are dedicated to mothers who have only children between 0 and 3 years of age, and the following three other columns focus on mothers who have at least one child between 0 and 3 years of age. The columns on active labour force participation and high part-time rate demonstrate that the effects are centred on the years 2009–2011. Before and after this period, the effects are not particularly significant, and the effects on active labour force participation often change in sign. The reform has therefore taken some years to produce significant effects on the labour supply of mothers, and these effects dwindled after the last years in which data on childcare expansion were collected.^13^ Overall, it appears that the reform has not increased female labour force participation for inactive mothers. For women who already worked, however, the reform increased the probability of working at high part-time participation rates. The average effect of 2 percentage points is significant only for mothers who may also have older children. This increase did not affect full-time participation. This significant change is therefore attributable to women who increased their working hours.^14^ The impact of the reform explains 19% of the overall increase in high part-time work registered between 2002 and 2014.

**Table 3:** Difference-in-differences estimates of the impact of the reform on maternal employment

	Only 0–3			Also 4–15		
	Active	High part-time	Full-time	Active	High part-time	Full-time
2003	− 0.01	0.01	0.00	− 0.01	0.00	0.01
2004	− 0.03	0.00	0.00	− 0.01	0.02	0.01
2005	− 0.03	0.03	− 0.02	− 0.04	0.01	0.00
2006	0.02	0.04	0.01	0.00	0.01	0.01
2007	− 0.04	0.04	0.00	− 0.04	0.03	0.00
2008	0.00	0.02	− 0.01	− 0.02	0.02	− 0.01
2009	0.03	0.05**	− 0.01	0.03	0.07**	0.00
2010	0.01	0.02	− 0.01	0.02	0.02	− 0.02
2011	0.02	0.04	0.02	0.00	0.04*	0.02
2012	− 0.03	− 0.02	0.02	− 0.04*	0.00	0.01
2013	− 0.02	− 0.01	0.00	− 0.03	0.00	− 0.01
2014	− 0.01	0.01	− 0.01	− 0.03	0.01	− 0.01
Average effect	− 0.01	0.02	− 0.00	− 0.02	0.02*	0.00
t-statistic	(− 0.28)	(1.11)	(− 0.14)	(− 1.00)	(1.72)	(0.13)
N	23,357	23,357	23,357	43,519	43,519	43,519

Source: Swiss Labour Force Survey 1992–2014

The sample is restricted to mothers between 20 and 50 years of age who have at least one child between 0 and 3 years of age. Controls include age, education, marital status, nationality, number of children and income of other family members. In all specifications, clustered standard error at the cantonal level and cantons and years fixed effects are used. *T*-statistics are reported in parentheses above the number of observations. ** and * denote statistical significance at 0.01 and 0.05 levels, respectively

Table 4 shows average effects of the impact of the reform according to the same socio-demographic characteristics illustrated in Table 1. The results are found to change according to the educational level of mothers. Similar to what was found by Stadelmann-Steffen (2007), childcare places are found to stimulate labour force participation among mothers with an upper-secondary educational level. In addition to this, the effects of the reform are registered only for women who are married or cohabitating, rather than for single mothers. As seen in Table 3, the effect is significant only for mothers with more than one child. The increase in the number of working hours, however, does not affect mothers with large families. To summarise, the increase in high part-time work concerns women with the most common socio-demographic characteristics, namely, those who have completed upper-secondary education, are married or cohabitating and have two children.

**Table 4:** Difference-in-differences estimates of the impact of the reform on maternal employment by different individual and household characteristics

	Active	High part-time	Full-time
Disaggregation by educational levels			
Lower-secondary	0.01	0.01	− 0.01
	(0.17)	(0.27)	(− 0.20)
	6687	6687	6687
Upper-secondary	− 0.01	0.05**	0.02
	(− 0.35)	(2.62)	(1.52)
	24,446	24,446	24,446
Tertiary	− 0.05	− 0.02	− 0.03**
	(− 1.32)	(− 0.53)	(− 2.31)
	12,386	12,386	12,386
Disaggregation by marital status			
Married or cohabitating	− 0.02	0.02*	0.00
	(− 1.04)	(1.80)	(0.13)
	41,869	41,869	41,869
Single mothers	− 0.08	− 0.02*	− 0.04
	(− 0.89)	(− 1.96)	(− 0.52)
	1650	1650	1650
Disaggregation by number of children			
1 child	− 0.01	0.02	− 0.01
	(− 0.31)	(0.89)	(− 0.21)
	16,845	16,845	16,845
2 children	− 0.02	0.02*	0.00
	(− 0.65)	(1.72)	(0.20)
	18,937	18,937	18,937
3 or more children	− 0.03	0.00	0.00
	(− 0.62)	(0.06)	(0.21)
	7737	7737	7737

Source: Swiss Labour Force Survey 1992–2014

The sample is restricted to mothers between 20 and 50 years of age with at least one child between 0 and 3 years of age. Depending on the disaggregation, controls include age, education, marital status, nationality, number of children and income of other family members. The difference-in-differences corresponds to a model with controls, cantons and years fixed-effects and clustered standard errors. *T*-statistics are reported in parenthesis above the number of observations. ** and * denote statistical significance at 0.01, 0.05 levels, respectively

### Robustness checks and paternal employment

In this section, the stability of the results is checked by several robustness checks. These checks include a test on the socio-demographic characteristics of mothers in treated and control groups, an analysis of fertility rates, a test on the displacement of other forms of care, an analysis of paternal employment, an analysis over a restricted number of years, and an analysis over a restricted number of municipalities.

The first check tests whether there were changes in the socio-demographic characteristics between the two groups of cantons after the introduction of the reform. Some municipalities tried to attract highly qualified foreign workers through an expansion of childcare services, and if they succeeded, a change in the socio-demographic characteristics of mothers should at least partly signal this effect. Descriptive evidence is provided in Table 2, where many socio-demographic characteristics seem to differ over time between the control and the treated group. A formal test is needed, however, to see whether these differences are significant. This test is performed through a DiD similar to Eq. (1), where the dependent variable, instead of being *Y*_*it*_, is one of the *X*_*it*_. Table 5 shows that all the coefficients linked with the socio-demographic characteristics *X*_*it*_ are not significant. It can thus be concluded that the composition of mothers residing in the two groups of cantons was not influenced by the reform. As a cautionary note, however, it is important to stress that although the composition of the treatment and control groups has not changed in a systematic way with respect to these observed socio-demographic characteristics, there may be unobserved heterogeneity that could bias the results. Overall, this robustness check highlights that the results from Table 3 are robust to international and inter-cantonal mobility with respect to observed characteristics. At least at a cantonal level, the efforts of some municipalities to attract highly qualified workers through childcare services seem to have been unsuccessful. However, it is important to note that cantonal aggregation might hide the effects of some successful municipalities in big cantons.

**Table 5:** Difference-in-differences estimates of the impact of the reform on the socio-demographic characteristics of mothers

Lower secondary educ.	Upper secondary educ.	Tertiary educ.	Married or cohabiting	No. of children 0–3	No. of children 4–15	Swiss nationality	Age	Income of other family members
− 0.01	0.03	− 0.02	0.02	0.00	− 0.02	0.00	− 0.19	− 1.19
(− 0.35)	(0.77)	(− 0.99)	(0.95)	(0.26)	(− 0.88)	(0.23)	(− 0.80)	(− 0.19)
43,519	43,519	43,519	43,519	43,519	43,519	43,519	43,519	43,519

Source: Swiss Labour Force Survey 1992–2014

The sample is restricted to mothers between 20 and 50 years of age with at least one child between 0 and 3 years of age. Depending on the disaggregation, controls include age, education, marital status, nationality, number of children and income of other family members in 1000 CHF. The difference-in-differences corresponds to a model with controls, cantons and years fixed effects and clustered standard errors. *T-*statistics are reported in parenthesis above the number of observations

If fertility increased with the activity rate of women, then it would be difficult to compare the same set of women over time. The effects of the reform would then reflect changes in employment and in fertility, as illustrated by Nollenberger and Rodríguez-Planas (2015). The normal hypothesis under this particular reform is that women in treated cantons decided to have more children because they could rely on external childcare and did not have to choose between a family and a career. To test this, the same DiD model is employed in a second robustness check. The main dependent variable here is the likelihood of having a child less than 1 year old. Table 6 shows that women in treated cantons do not have a significantly higher or lower fertility rate than women in control cantons. When performing these regressions with yearly estimates, significant increases in fertility were, however, reported in 3 years: 2006, 2007 and 2012.

**Table 6:** Difference-in-differences estimates of the impact of the reform on fertility rates, use of institutional childcare and paternal employment

Fertility	Institutional childcare use	Paternal participation	Paternal high part-time	Paternal full-time
− 0.00	0.02	0.01	0.00	0.01
(− 0.12)	(0.77)	(0.52)	(0.29)	(0.54)
43,519	19,017	39,416	39,416	39,416

Source: Swiss Labour Force Survey 1992–2014

The sample is restricted to mothers between 20 and 50 years of age with at least one child between 0 and 3 years of age. Controls include age, education, marital status, nationality, number of children and income of other family members. The difference-in-differences corresponds to a model with controls, cantons and years fixed effects and clustered standard errors at the cantonal level. *T*-statistics are reported in parenthesis above the number of observations

**Fig. 4:**
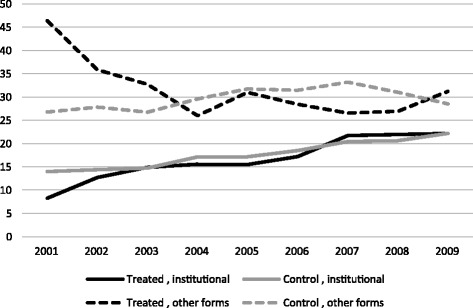
Evolution of childcare use by type of care in treated and control cantons. Source: Swiss Labour Force Survey 2001–2009. The sample is restricted to mothers between 20 and 50 years of age with at least one child between 0 and 3 years of age

A legitimate claim could also be made that users of other forms of childcare switched to institutional childcare^15^ once childcare subsidies became effective. To test whether or not this was the case for the third robustness check, the use of other childcare services is compared with the use of formal childcare. Figure 4 shows that the use of other forms of care declined in treated cantons until 2004. The hypothesis that institutional care substituted for other types of care therefore appears more likely prior to the introduction of subsidies. It is also evident that, in both groups of cantons, the expansion in childcare use between 2001 and 2009 concerned mainly institutional childcare. This similar childcare use between treated and control cantons is confirmed by a DiD model on the use of institutional childcare over other forms of childcare, which did not yield a significant coefficient (Table 6). Even if comparable data ended in 2009, what can be drawn from this short analysis is that, until 2009, institutional care was unlikely to have displaced other complementary forms of care.^16^ Another quick conclusion that Fig. 4 may suggest is that the large expansion in childcare places in treated cantons has been ineffective, because mothers in those cantons have not increased their use. Even if it is true that the use of institutional care has not increased more in treated cantons compared to control cantons, Fig. 4 may hide an intensive margin effect on the amount of hours of care used. This interpretation supports the main results regarding maternal employment. Childcare use is not reported at full-time rates in the SLFS, and this means that Fig. 4 represents only the number of mothers (weighted by number of children) who regularly use an external service for childcare. As ‘regularly’ may refer to once a week or once a month, and not every hour of every day of the week, this figure does not take into account frequency of use. When exploited at part-time rates, a single childcare place could be used by more than one mother during the same week.^17^ For this reason, Figs. 1 and 4 are not perfectly comparable. Similar to the insignificant effect on female labour force participation, the increase in the number of mothers that use institutional childcare in treated cantons has not been larger than the increase in control cantons. In this sense, the greater expansion in childcare services in treated cantons has not boosted the number of women who are active on the labour market. Nevertheless, it has allowed more women to work longer hours or more days per week.

With the increasing engagement of men in childrearing, it is also possible that an expansion of childcare places changed employment patterns among men. The male labour force participation rate is stable approximately 95% and has not changed over time. Unlike for mothers, there is no remarkable difference in male participation between cantons or between fathers of children of different ages. Any DiD regression applied to fathers with yearly estimates yielded insignificant results. It can thus be concluded that the expansion of childcare places affected only female employment.

To control for the stability of the results, two tests are performed based on the introduction of almost simultaneous policies (i.e., maternity leave and legal enforcement of after-school care), and two tests based on the expansion of childcare. The test on maternal employment and the enforcement of paid maternity leave is based on DiDs that isolate cantons that introduced the policy in a specific year from those that implemented it previously (for maternity leave) or those that never implemented it (for the enforcement of after-school care). For maternity leave, the cut-off year is 2005,^18^ whereas the cut-off is 2009 for the legal enforcement of after-school care.^19^ The results displayed in Table 7 show that those cantons that introduced paid maternity leave in 2005 do not register significantly different behaviour in terms of maternal employment. This is likely due to both the late introduction of the reform—which happened when all but four cantons had already established paid maternity leave—and the small number of weeks of paid maternity leave (Baker and Milligan 2008). The second test shows that the enforcement of after-school care did not produce significant effects on mothers with at least one child between 0 and 3 years of age. These mothers may be jointly affected by after-school care, because they possibly also have older children. With the most appropriate sample, Felfe et al. (2016) found that the effect was indeed significant for those mothers with at least one child between the ages of 4 and 12. With a sample of mothers of younger children, this test shows only that there are no confounding effects of the legal enforcement of after-school care on the main effect found for the expansion of childcare.

**Table 7:** Difference-in-differences estimates of the impact of the enforcement of maternity leave on maternal employment

Test	Active	High part-time	Full-time
Paid maternity leave	− 0.01	− 0.03	− 0.00
	(− 0.50)	(− 1.64)	(− 0.12)
	19,744	19,744	19,744
Enforcement of after-school care	0.00	0.02	0.01
	(0.07)	(0.91)	(0.74)
	34,431	34,431	34,431

Source: Swiss Labour Force Survey 1992–2014

The sample is restricted to mothers between 20 and 50 years of age with at least one child between 0 and 3 years of age. Controls include age, education, marital status, nationality, the number of children and income of other family members. The difference-in-differences corresponds to a model with controls, cantons and years fixed effects and clustered standard errors at the cantonal level. *T*-statistics are reported in parenthesis above the number of observations

Other tests with restrictions on the expansion of childcare services are reported in Table 8. The first test of the table restricts the years of the analysis, and the second test addresses endogeneity, limiting the analysis to mothers who live in areas with similar labour markets and similar preferences for family policies.

**Table 8:** Difference-in-differences estimates of the impact of the reform on maternal employment using alternative controls

Test	Active	High part-time	Full-time
1992–1998, 2006–2012	− 0.01	0.03*	0.00
	(− 0.45)	(1.97)	(0.23)
	25,371	25,371	25,371
Local labour markets	− 0.03	0.03	0.01
	(− 1.45)	(1.63)	(0.78)
	33,788	33,788	33,788

Source: Swiss Labour Force Survey 1992–2014

The sample is restricted to mothers between 20 and 50 years of age with at least one child between 0 and 3 years of age. Controls include age, education, marital status, nationality, number of children and income of other family members. The difference-in-differences corresponds to a model with controls, cantons and years fixed effects and clustered standard errors at the cantonal level. T-statistics are reported in parenthesis above the number of observations. * denotes statistical significance at the 0.05 level

The results are quite robust to alternative specifications. The restriction to years that are not close to the introduction of the subsidies causes the coefficient to increase by 1 percentage point, but does not change its significance level. It is notable, however, that the *T*-statistic of the coefficient increased. The second test yields insignificant results. Although it does not reach the minimum significance level of 0.10, the coefficient for high part-time rates does not change dramatically in magnitude from the basic specification, and equals the coefficient found in the first alternative specification of Table 7. This indicates that previous coefficients were not severely biased due to endogeneity.

### Results in perspective: effects, subsidies and policies

Many studies have addressed the issue of the effects of an expansion in childcare services on female labour supply in different countries. To my knowledge, however, the results of these studies are dependent on the sample selected, years analysed, country studied and methodology chosen (see Bettendorf et al. 2015 for a review). Unlike the studies on Scandinavian countries presented in the introduction, studies on countries in which childcare services are not extremely well developed find a strong increase in female labour supply associated with the creation of childcare places. This is the case in Argentina, for instance, where with every 100 new places created in childcare facilities, 13 mothers began working (Berlinski and Galiani 2007; Berlinski et al. 2011), and in Spain, where reforms granted public places in childcare facilities for 70% of children and additional private places for another 20% of children (Nollenberger and Rodríguez-Planas 2015).

Switzerland is particular in this respect, because childcare coverage is underdeveloped from an international perspective, and coverage remained quite limited even after the reform. Even though childcare provision is not homogeneous across cantons, those cantons that expanded childcare more rapidly between 2002 and 2012 managed to cover only 17.4% of children on average in both private and public childcare facilities. This expansion has only modestly affected the labour market, even considering that female labour force participation is relatively high. In Switzerland, between 1992 and 2014, there was no significant change in active female labour force participation as a result of the expansion of childcare places. However, a significant change was found in high part-time employment, defined as at least a part-time rate of more than 50%. The increase was indeed small, and accounted for 2 percentage points. Estimates increased to 3 percentage points in a robustness check that excludes the years close to the reform. These effects are in accordance with those found by Felfe et al. (2016) for after-school care in German-speaking cantons. The results in this major linguistic region of Switzerland pointed to an expansion of full-time employment, defined as more than 36 working hours per week. This evidence suggests that the results can also be extended to pre-school care and to non-German-speaking cantons. Following the definition of part-time work adopted by the Swiss Labour Force Survey, this paper suggests that changes in employment concern mainly high part-time rates.

Regarding socio-demographic groups, this paper supports the results on education found by Stadelmann-Steffen (2007). In Switzerland, the effects of childcare are found to be stronger for mothers with upper-secondary education, but contrary to results from other countries (Blau and Tekin 2007; Goux and Maurin 2010; Fitzpatrick 2010, 2012), married or cohabitating mothers benefit more from childcare than single mothers. This result could be due to the fact that this study takes into account both private and public childcare places. The expansion of private childcare places often does not interest single mothers, because prices are too high. The same argument regarding prices could also be used for large families. Moreover, in Switzerland, single mothers are given priority for subsidised places and are thus less likely to remain on the waiting lists. As found by Nollenberger and Rodríguez-Planas (2015), the reform was more effective for mothers with two children, who may decide to resume work more easily than mothers who desire to have more than one child. These results are found to affect only female employment, because male employment was not altered by the availability of childcare. This is similar to the determination made by Felfe et al. (2016).

Regarding the amount of spending for the reform, the results for working mothers in Switzerland are lower than what was found in the Netherlands by Bettendorf et al. (2015). An additional subsidy of 2 billion euros in the Netherlands increased female labour force participation by 2.3 percentage points and hours worked by mothers by 6.2%. This translates into 90,000 euros per additional full-time equivalent position created by the reform (Bettendorf et al. 2015). In Switzerland, total investment between 2003 and 2019 is estimated at 440 million Swiss francs for both pre-school and after-school care between 2003 and 2019. The resources allocated to early childcare in Switzerland are therefore considerably lower than those granted by the Dutch government; thus, it should not be surprising that the effects on maternal employment are not remarkable. The generosity of the subsidy may play a role, yet this remains an interesting comparison because these two countries have similar rates of female labour force participation.

Childcare reforms were accompanied by another family policy, namely, the legal recognition of maternity leave and the enforcement of after-school care in some cantons. A similar joint reform of childcare and maternal leave occurred in Germany in 2007 (see Geyer et al. 2015). Despite this similarity, the effects of these policies in Switzerland, mainly on childcare expansion, are minimal compared to the extensive increase in maternal employment found in Germany (7 percentage points, both in part-time and in full-time employment). A similar policy setting is also present in Canada. Confirming results from other countries, childcare subsidies there had a greater impact on the gender employment gap than parental leave (Milligan 2014).

## Conclusions

Childcare expansion is a family policy that many researchers consider non-distortive in terms of gender-oriented discrimination (Hegewisch and Gornick 2011). Childcare has often been proven to produce other desirable effects, such as a fairer distribution of household duties between couples (Epple et al. 2014), and sometimes even higher fertility rates (Del Boca 2002; Bonoli 2008; Haan and Wrohlich 2011). The effects of childcare speak not only to the issue of female labour supply but also to the wellbeing of children. It is indeed found that children who use institutional childcare services benefit from a higher quality of childcare (Haeck et al. 2015) and are better able to develop their cognitive abilities independently of the educational level of their parents (Havnes and Mogstad 2011a; Crettaz and Jacot 2014; Felfe et al. 2015). The drawback of this powerful policy, however, is the high cost to governments for such an intervention. Given the significant share of resources at stake, this important issue is relevant to those making decisions at the highest levels. The Swiss federal system offers a very good experimental setting in which to test these policy outcomes, as each of the 26 cantons has broad freedom of action in terms of policy implementation. In this context, the institutionalisation of maternal leave and the expansion of childcare caused a small but significant increase in female high part-time employment. Compared to other European countries, this increase was indeed small, although in line with the small expansion of childcare places (compared to Spain), the amount of subsidies allocated (compared to the Netherlands) and the short duration of parental leave (compared to Germany).

It is important to state that this expansion in childcare did not particularly benefit vulnerable groups, such as low-educated mothers, single mothers or large families. This is likely due to the high cost of institutional childcare in Switzerland. To better test this assumption, a cost-benefit approach should be performed through a labour supply model (as in Müller and Wrohlich 2015), and further research is needed in this regard.

As of the present, it is interesting to note that neither governmental support nor social norms have increased paternal involvement in childcare roles.

Finally, it is important to point out that these results rely on a new collection of data at the cantonal level. Despite being a new source of information, the data can be considered limited, given that it is based on the averaging of childcare among all municipalities within a canton. Although the evidence retrieved from a set of German-speaking municipalities supports the results found in this study (Felfe et al. 2016), whenever possible, new data collections on childcare should focus on the municipal level in order to truly capture the socio-economic diversity of Switzerland.

## Appendix

**Table 9:** Child-care coverage across cantons, 2002 and 2012

Canton	2002	2012	Expansion in %
Zürich	11.71	19.42	65.83
Bern	12.13	16.64	37.17
Luzern	5.98	7.36	23.05
Schwyz	4.15	5.52	33.14
Nidwalden	2.68	4.70	75.24
Glarus	5.33	10.26	92.34
Zug	10.11	14.61	44.58
Fribourg	6.60	10.24	55.14
Solothurn	6.12	10.84	77.25
Basel-Stadt	18.43	48.56	163.53
Basel-Landschaft	4.80	17.77	269.95
Schaffhausen	7.45	19.06	156.04
Appenzell A.Rh.	2.61	9.80	274.75
Appenzell I. Rh.	0.87	0.90	3.59
St. Gallen	2.57	6.16	139.73
Graubünden	2.00	8.42	321.57
Aargau	7.48	8.98	20.01
Thurgau	4.54	8.39	84.89
Ticino	2.88	11.53	300.87
Vaud	16.37	29.47	80.04
Valais	7.45	15.59	109.32
Neuchâtel	19.50	27.90	43.10
Genève	15.41	23.52	52.66
Jura	3.22	7.32	127.07

Sources: Ravazzini et al. (2016), 2012 cantonal statistics for Bern, Neuchâtel and Vaud

## Footnotes

1 This trend cannot be entirely generalised for Scandinavian countries, because Danish childcare prices and availability, for instance, are highly relevant for female employment (Simonsen 2010).

2 In Switzerland in 2012, only 7.6% of part-time working mothers would have preferred a full-time contract, whereas 41.6% of full-time working mothers would have preferred a part-time contract. Similar figures were produced with the ISSP in 1997 (Stier and Lewin-Epstein 2003). This indicates that preferences have not changed over time.

3 According to the OECD Family Database, childcare fees are 67.3% of the average wage. This is the highest proportion among the 36 OECD countries. For middle-class two-income families, this corresponds to 23.6% of net household income. Switzerland can be ranked together with other liberal countries with a proportional fee for childcare 2.4 times higher than in Germany or France and almost 9 times higher than in Austria.

4 Statistics about the number of places provide information regarding what the Swiss Federal Statistical Office has defined as “institutional care”. Independently from public funding, institutional care includes pre-school institutions, after-school institutions, nursery schools and associations of trained personnel specialising in day-care services for children.

5 The stratification of the survey is based on cantons only in 1995 and since 2009 onwards. In all other years, the stratification is limited to macro regions (Nuts II). Nuts II include the following seven macro-regions: Vaud, Valais and Genève; Bern, Fribourg, Solothurn, Neuchâtel and Jura; Basel-Stadt, Basel-Landschaft and Aargau; Glarus, Schaffhausen, Appenzell A.Rh., Appenzell I. Rh., St. Gallen, Graubünden and Thurgau; Luzern, Uri, Schwyz, Obwalden, Nidwalden and Zug; Zürich; and Ticino.

6 The only two cantons missing are Uri and Obwalden.

7 Half of the cantons have information about 1 or 2 years before or after 2002, whereas six cantons have information on childcare places in 2010, but not in 2012.

8 A collection of data on preschool and after-school care at the municipal level was performed by Stern et al. (2013) in 2009–2010. No such previous collection was performed, and this prevents the use of these data in an application with a quasi-experimental design using the number of childcare places. Felfe et al. (2016) employed these data in a two-stage-least-square estimator instrumenting the variation in the cantonal reforms of after-school care. Cantonal reforms were also used in a difference-in-differences framework that did not look at the after-school supply.

9 The sample has been restricted to this age range for fertility reasons.

10 The treated group comprises the cantons of Basel-Stadt, Basel-Landschaft, Schaffhausen, Appenzell A.Rh., St. Gallen, Graubünden, Ticino, Vaud, Valais and Jura. The control group comprises the cantons of Zürich, Bern, Glaris, Zug, Fribourg, Solothurn, Appenzell I. Rh., Thurgau, Neuchâtel, Genève, Luzern, Schwyz, Nidwalden and Aargau. More details are provided in Table 9.

11 These thresholds (i.e. 50 and 90%) correspond to national distinction between low and high part-time rates and between high part-time rates and full-time work. A continuous variable for the number of hours worked was not available in all years of the SLFS. In a standard week of 40 working hours, high part-time corresponds to between 20 and 36 h of work.

12 Similar voting preferences refer to intended votes that do not differ by more than 5 percentage points.

13 Data on childcare expansion are available up to 2012, and after this year, other cantons in the control group may have expanded their childcare supply. The exclusion of 2013 and 2014 does not change the magnitude of the coefficients or the significance level of the coefficients. A test without these 2 years is also shown in Table 7.

14 If mothers who work at high part-time rates are isolated and compared only to mothers who work at low part-time rates, then the effect becomes larger (5 percentage points) and highly significant. This specification (not displayed in Table 3) may however suffer from endogeneity.

15 After-school care is not treated in order to provide better comparability with Fig. 1. Even if at different levels, time trends in the use of institutional and other types of care are also representative for after-school services.

16 Official statistics provide some figures regarding the use of childcare from the 2013 Survey of Income and Living Conditions (SFSO 2016). From these figures, it appears that 73.8% of children up to 3 years of age are taken care of by an external carer. Of these, 17.6% are exclusively cared for by a non-institutional entity such as grandparents, nannies and daily mothers, and 56.3% by an institutional entity. Thus, by 2013, the majority of young children were at least partly cared for in a childcare facility.

17 According to the Federal Social Insurance Office, only 12% of all children using childcare services use childcare facilities 5 days per week (35% of children, for instance, use the service 2 days per week, and 20% use it only 1 day per week). Of all children using childcare at least 1 day per week, 69% use a daily service, 30% use the service only for the first or the second half of the day, and 2% of children use an hourly service.

18 According to Guillet et al. (2016), the cantons that introduced maternity leave in 2005 are Bern, Graubünden, Scaffausen and Uri. We exclude from the comparison those cantons that gradually introduced paid maternity leave between the early 1990s and 2005. This DiD therefore compares Bern, Graubünden, Scaffausen and Uri to cantons that already granted paid maternity in the early 1990s (Appenzell A.Rh., Basel-Stadt, Jura, Neuchâtel, Schwyz, Ticino, Thurgau, Valais and Vaud).

19 According to Felfe et al. (2016), the cantons that introduced enforced after-school care by 2009 are Bern, Solothurn and Zurich. Geneva already introduced it in the 1990s and is excluded from the comparison. Basel City, Graubünden, Luzern, Neuchâtel, and Schaffhausen introduced it between 2010 and 2015, and hence, they are also excluded from the comparison. This DiD compares Bern, Solothurn and Zurich with all cantons that never introduced the enforcement of after-school care before 2015.
